# Genomic characterization of two community-acquired methicillin-resistant *Staphylococcus aureus* with novel sequence types in Kenya

**DOI:** 10.3389/fmed.2022.966283

**Published:** 2022-09-26

**Authors:** John Njenga, Justin Nyasinga, Zubair Munshi, Angela Muraya, Geoffrey Omuse, Caroline Ngugi, Gunturu Revathi

**Affiliations:** ^1^Department of Pathology, Aga Khan University Hospital, Nairobi, Kenya; ^2^Department of Medical Microbiology, School of Biomedical Sciences, Jomo Kenyatta University of Agriculture and Technology, Nairobi, Kenya; ^3^Center for Microbiology Research, Kenya Medical Research Institute, Nairobi, Kenya; ^4^Department of Biomedical Sciences and Technology, Technical University of Kenya, Nairobi, Kenya; ^5^Pan African University – Institute of Science, Technology, and Innovation (PAUSTI), Nairobi, Kenya; ^6^United States Army Medical Research Directorate Africa, Nairobi, Kenya

**Keywords:** CA-MRSA, WGS, novel sequence, AMR, Kenya

## Abstract

*Staphylococcus aureus* is a clinically important bacteria with high antimicrobial resistance (AMR) challenge globally. The emergence of methicillin-resistant *Staphylococcus aureus* (MRSA) clones with unique sequence types have been identified in the community showing evidence that the epidemiology of MRSA globally is changing and requires continual surveillance. We utilized whole genome sequencing to characterize two community acquired-MRSA (CA-MRSA) strains isolated from wound swabs from community-onset infections in two health facilities in Kenya. The two strains belonged to multilocus sequence type (MLST) sequence type (ST) 7460, and ST 7635. The resistance genes detected showed that the novel STs are carriers of clinically relevant resistance genes. Linezolid and mupirocin resistance was observed, yet mupirocin is not commonly used in the country. Mutations within resistance genes were also detected and the pathogenicity toward the human host matched various pathogenic global *S. aureus* families, e.g., *S. aureus* subsp. *aureus* USA300. Multidrug efflux transporters, important in antimicrobial resistance including restriction enzymes type I and type IV were detected. Plasmids identified showed similarities with the plasmids in other clinically significant non-staphylococcal species, such as *Pseudomonas aeruginosa, Escherichia coli, Morganella morganii*, and *Enterococcus faecium*. Both STs belong to clonal complex 8 (CC8) which is the most successful MRSA clone in Kenya. Spa type t30 to which ST 7635 belongs has not been reported in the country. The results of this study further highlight the need for epidemiological studies to reveal circulating strains and antimicrobial resistance spread between hospitals and the community. The genomic research highlights resistance to anti-staphylococcal broad-spectrum antimicrobials not used frequently in the country, jeopardizing successful MRSA treatment since most health facilities do not perform genotypic resistance tests for routine patient management. Preliminary insights into unidentified STs of CA-MRSA in Kenya show the need for molecular epidemiological surveillance studies to further understand the diversity of *S. aureus* in Africa.

## Introduction

*Staphylococcus aureus* (*S. aureus*), a commensal in humans and an abundant bacteria of the skin microbiome, is a major pathogen that causes a wide range of clinical infections (1, 2). *S. aureus* has thrived as a human pathogen due to its ability to infect any tissue, for example, the skin, where it adapts within the environment and transitions to cause infection (3). The growing prevalence of community-associated methicillin-resistant *Staphylococcus aureus* (CA-MRSA) presents a clinical challenge in the management of serious infections worldwide (4).

The emergence of methicillin-resistant *Staphylococcus aureus* (MRSA) clones with unique sequence types have been identified in the community, suggesting evolution within *S. aureus* (5). The epidemiology of MRSA worldwide is changing, and a rise in strains causing disease in populations with no associated risk factors in the community has been reported (6, 7). CA-MRSA has in the past two decades emerged as a clinically relevant pathogen causing skin and soft tissue infection (SSTI), which is cytotoxin driven with suggestions indicating that hypervirulent sequence types (STs) exist (7). MRSA has evolved over the years, from which clinically significant STs reported range from the 1960s. A good example is the ST250 MRSA-I published around 1965, consistent with ancient European types and the most widespread multidrug-resistant clone globally. ST1-MRSA-IV was reported in Australia in 1981 and the United States in 1990, and it is known to have high-level mupirocin resistance and multi-drug resistance characteristics. Novel strain ST2249-MRSA-III which was a multi-resistant clone was reported in 1973 and was important because gentamicin resistance first appeared in this strain. It is believed that 35.3% of ST239 chromosomes were inherited by this strain, and it’s also known to have caused the Australian epidemic in the early 1970s (8).

Community acquired-MRSA (CA-MRSA) has diverse clones which are dispersed in different clonal complexes, but only a few are genetically related (9). However, in Africa, *S. aureus* has not been the focus of research in the past despite the rich diversity that could have a significant impact, especially when studying the epidemiology of *S. aureus* infections (10). Knowledge of clones is important in medical practice because it allows antibiotic resistance studies to be done on each one of them. Notably, the molecular epidemiology of CA-MRSA shows poorly regulated sales of antibiotics in pharmacies, as well as empirical treatment, in the absence of laboratory investigation, contributes to the development of new MRSA clones with increased resistance to antibiotics (11).

Differentiating MRSA using whole genome sequencing (WGS) is important to characterize strain diversity, and understand evolution within MRSA (12, 13). In Kenya, a developing country, various sequence types of both MRSA and methicillin-susceptible *Staphylococcus aureus* (MSSA) from both hospitals and community (some being global MRSA strains), have been identified and they include; ST 22, ST8, ST39, ST1290, ST241, ST1, ST5, ST8, ST152 from Thika (Kiambu county), Kericho (Kericho county), Nairobi (Nairobi county) and Kisumu (Kisumu county) (14). This is important in studying evolution in MRSA especially the co-existence between CA-MRSA and hospital-associated MRSA (HA-MRSA) and the emergence of new resistance as a result of co-existence. It also is essential in studying key genomic mutations which have a public health significance and impact, such as new insertion sequences (IS) that affect virulence and pathogenicity toward a human host. For example, symptoms presented by patients infected with MRSA strain with genomic mutations, such as IS256 in USA500, whose outcome causes increased virulence and heightened pathogenicity and cytotoxicity, may present differently in a different geographical setting. The purpose of this study was to characterize the two novel ST 7460 and ST 7635 by WGS, to determine antimicrobial resistance profiles, spread, virulence characteristics, and genetic diversity. Molecular typing of the novel isolates by WGS has enabled track spread of MRSA by MLST and has further provided an in-depth insight into MRSA evolution. The two isolates were from community-onset staphylococcal infection, from Kiambu and Nyeri County health facilities, which are AMR surveillance sites in Kenya. Characterization of the novel sequence types ST 7460, and ST 7635, further highlight the need for medical laboratories to perform molecular diagnosis, including antibiotic resistance gene detection and resistance to specific antibiotics, due to other mechanisms. Molecular diagnostic testing is infrequently performed in medical laboratories and less so in resource-limited countries. Performing molecular diagnosis will guide clinicians on how best to manage their patients.

## Materials and methods

### Study isolates

Two CA-MRSA isolates from a surveillance study of SSTIs in five AMR surveillance sites in Kenya were characterized by WGS and had novel STs. A cross-sectional study design was employed, and a simple random sampling method was used. The samples were collected from outpatient departments of the selected facilities, within one year. All recruited patients did not have a clinical history/procedure within the past year and were not on antibiotic treatment for the SSTI. Patients recruited had clinical features of wound infection which brought them to the clinics. They had no history of international travel (never traveled abroad). Bacteria isolation was done using blood agar and identification was performed using standard microbiology procedures (15), antimicrobial susceptibility was performed guided by the clinical laboratory standards institute (CLSI) (16) using Vitek Compact 2 (bioMerieux Inc), an automated bacteriology system that identifies the bacterial pathogen and tests for a panel of 20 antibiotics providing breakpoint susceptibility by providing 3 minimum inhibitory concentration (MIC) cut-off values at Resistance, Intermediate and Susceptible points.

### Whole genome sequencing, genome assembly, and annotation

DNA library was prepared using the Collibri™ PCR-free ES DNA Library Prep Kit (Thermofisher, Massachusetts, USA) and quantification was done using Collibri™ Library Quantification Kit (Illumina, Inc., California, USA). Sequencing was performed on the Illumina MiSeq platform. Quality assessment for raw data was done by *fast QC* and data was trimmed using Trimmomatic (v 0.36) (17), and assembled using Shovill (v3.0) (18). QUAST (v 5.0) (19) and BUSCO (v 5.3.2) (20) were used to perform quality assessment for genome assemblies and annotations. SCC*mec*Finder and MLSTFinder, both hosted within the Centre for Genomic Epidemiology (CGE)^1^ were used for SCC*mec* and MLST typing, respectively. Further, genome assemblies were run in PubMLST^2^ (21) for Multi-locus sequence typing. Spa typing [Based on the sequence of the X polymorphic region of the A protein (spa) where the constitution of the X region is a variable number of 24-bp repeats flanked by well-conserved sequences/regions] was performed on the CGE platform using the Spatyper tool.^3^ Further, the contig sequences were used to query for spa types on the web-based SpaTyper^4^ and repeat sequences verified on the Ridom Spa Server^5^ (22).

**FIGURE 1:**
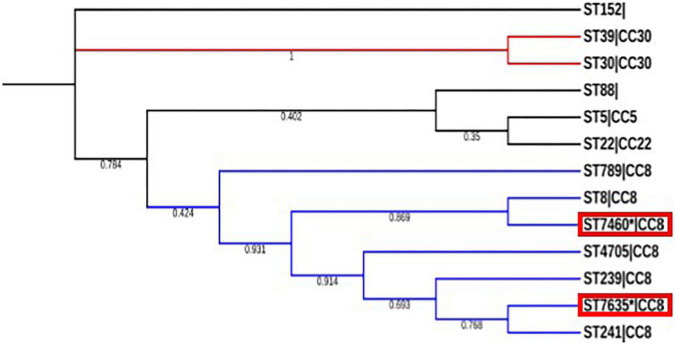
Genetic relatedness of the two novel sequence types to other sequence types identified in the country. The novel sequence types (ST7460 and ST7635) are highlighted in red boxes. The tree topology was based in 1000 iterations with bootstrap support expressed as probability values. The blue branches highlighted CC8 to which ST 7460 and ST 7635 belong.

Resfinder and virulencefinder (both available at the center for genomic epidemiology CGE), were used to identify acquired antibiotic resistance/mutations within resistance genes and acquired virulence, respectively. PathogenFinder^6^ was used to predict pathogenicity toward a human host. Mobile genetic elements (MGE)^7^ based on top hits, were used to identify mobile genetic elements and their relation to antimicrobial resistance genes and virulence factors. Plasmid identification and homology parameters were set at 99–100%. A comprehensive antibiotic resistance database (CARD)^8^ (23) was also used to identify resistance genes and resistance mechanisms. Assembled contigs were also submitted to the bacterial whole genome sequence database^9^ (24) for whole genome sequence typing and source tracking. To add some epidemiological context to the novel STs we performed a phylogenetic analysis of 11 other reported MRSA strains from previous studies in the country (13, 25, 26). Concatenated sequences were retrieved from the PubMLST database and a multiple sequence alignment was performed using the Multiple Alignment with Fast Fourier Transform (MAFFT). A maximum likelihood tree with a generalized time-reversible model was constructed for the 12 local MRSA STs using the Molecular Evolutionary Genomic Analysis (MEGA) (25). Version 11.0. 11 software with bootstrapping parameter set at 1000. The tree was refined using the Interactive Tree of Life (iTOL v. 6.5.6) (Figure 1). The reference genome used was N315_BA000018_ST5.

## Results

### Characterization of isolates with novel alleles

Isolate SA004 contained a novel arcC allele number 757 and assigned novel ST 7460 and isolate SA002 with novel allele glpf number 936 and assigned novel ST 7635. The isolates were identified as belonging to spa type t1476 and t30, respectively. The spa type of ST 7460 (t1476) and ST 7635 (t30) suggests that they may be members of the clonal complex 8 that constitutes ST8. Spa type t30 has not been reported in the country before. Interesting resistance was observed in the isolates, for instance, SA002 was only susceptible to 2 out of 13 antibiotics tested, while SA004 was only susceptible to 4 out of 13 antibiotics tested (Table 1). The antimicrobial susceptibility profiles of the two isolates with novel STs are in Table 1. SA002 was also found to be resistant to rifampin and linezolid phenotypically and had genes conferring rifampin resistance (*rpoB*), linezolid resistance (*cfr*), and mupirocin resistance (*mupA*) (Table 2). Various AMR gene families that are key to bacterial resistance were present in both SA002 and SA004 (Table 3). These include multidrug efflux transporters which are important in transporting antibiotics from inside to outside of bacterial cells (27–29) (Table 4).

**TABLE 1:** Resistance profiles of SA002 and SA004.

ANTIBIOTIC/ ISOLATE	Cefoxitin	Penicillin	Erythromycin	Clindamycin	Ciprofloxacin	Tetracycline	Vancomycin	Tigecycline	Linezolid	Rifampin	Trimethoprim/Sulfamethoxazole	Mupirocin	Gentamycin
SA002	R	R	R	R	R	R	*S*	*S*	*R*	*S*	*R*	*R*	R
SA004	R	R	R	R	R	R	*S*	*S*	S	*R*	*R*	*S*	R

S stands for susceptible and R stands for resistant.

**TABLE 2:** Resistance genes and mutation points detected within SA002 and SA004.

Class	Resistance gene(s)	Mutations points	Genotypic resistance (SA004)	Genotypic resistance (SA002)	Phenotypic resistance (SA004)	Phenotypic resistance (SA002)
Tetracyclines	*tet(K), tet(M)*	–	+	+	+	+
Beta-lactams	*mecA, blaZ*	pbp2:p.A606D, pbp2:p.A420V, pbp4:p.P220S, pbp4:p.L234H, pbp2:p.A285P, pbp2:p.E315A, pbp4:p.L234H	+	+	+	+
Macrolides	*erm(C), erm(A)*	–	+	+	+	+
Quinolones	*gyrA, parC*	gyrA:p.D402E, gyrA:p.E859V, gyrA:p.V598I, grlA:p.V694M, grlB:p.D530G, grlB:p.E471K	+	+	+	+
Carboxylic acid	*mupA*	–	–	+	No MIC	No MIC
Aminoglycoside	*aac(6’)-aph(2”)*	–	+	+	+	+
Sulfonamides	*dfrG, dfrB*	dfrB:p.I97T, dfrB:p.V72E, dfrB:p.V72E	+	+	+	+
Oxazolidinone	*cfr*	–	–	+	–	+
Glycylcycline	–	–	–	–	–	–
Glycopeptides	–	–	–	–	–	–
Rifamycin	*rpoB*	rpoB:p.S529L, rpoB:p.G767S	–	+	–	+
Amphenicol	*cfr*	–	–	+	No MIC	No MIC

MIC stands for minimum inhibitory concentration and the symbol (+) represents present and (–) represents absent.

**TABLE 3:** AMR gene families detected in SA002 and SA004.

AMR gene family	Organism	Antibiotic class target	Resistance mechanism	Antibiotic resistance ontology and some of the functions	
ATP-binding cassette (ABC) antibiotic efflux pump	SA002 SA004	Fluoroquinolone antibiotics, Cephalosporins, Penam, Macrolides, Aminoglycoside, Oxazolidinone antibiotic, Diaminopyrimidine antibiotic, Phenicol antibiotic, and Tetracyclines	Antibiotic efflux	mgrA	Indirectly regulates norA (fluoroquinolone resistance gene) and tet38 (tetracycline resistance gene) expression. The gene also modulates clumping and virulence of *Staphylococcus aureus* Indirectly regulates norA (fluoroquinolone resistance gene) and tet38 (tetracycline resistance gene) expression. The gene also modulates clumping and virulence of *Staphylococcus aureus*
Major facilitator superfamily (MFS) antibiotic efflux pump			Antibiotic efflux	mgrA,	
				arlS,	Activates expression of mgrA and also regulates oxacillin resistance in MRSA
				norC,	Induces nucleosome sliding Is also a non-coding RNA that regulates the transcription of rRNA
				Staphylococcus aureus LmrS	Is a multidrug efflux pump for lincosamide resistance protein in *S aureus*
Multidrug and toxic compound extrusion (MATE) transporter	SA002 SA004	Glycyclines and Tetracyclines	Antibiotic efflux	mepR,	Represses the expression of *S aureus* multidrug efflux pump gene, mepA
				mepA	Is an *S aureus* multidrug efflux pump
Small multidrug resistance (SMR) antibiotic efflux pump	SA002 SA004	Disinfecting agents	Antibiotic efflux	sepA	Facilitates biofilm maturation and intercellular adhesion.
Trimethoprim-resistant dihydrofolate reductase dfr	SA002 SA004	Diaminopyrimidine antibiotic	Antibiotic target replacement	dfrG	Plays a role in folate metabolism
APH (2”), AAC (6’)	SA002 SA004	Aminoglycoside antibiotics	Antibiotic inactivation	AAC(6’)-le-APH(2”)-la	Confers high-level gentamycin resistance and its presence jeopardizes the use of gentamycin and other aminoglycosides
Fosfomycin thiol transferase	SA002 SA004	Fosfomycin	Antibiotic inactivation	*Staphylococcus aureus* FosB,	Is a fosfomycin-inactivating enzyme that modifies an antibiotic into a compound that lacks bactericidal properties
Blaz beta-lactamase	SA002 SA004	Penam	Antibiotic inactivation	PC1 beta-lactamase (blaZ)	Enhances antibiotic resistance by catalyzing the hydrolysis of beta-lactams
Methicillin-resistant PBP2	SA002 SA004	Penam	Antibiotic target replacement	mecA	Gene responsible for methicillin resistance in *Staphylococci*
Erm 23S ribosomal RNA methyltransferase	SA002	Macrolide antibiotic, lancosamide antibiotic, streptogramin A antibiotic, streptogramin B antibiotic	Antibiotic target alteration	ErmA	Confer macrolide resistance in *S aureus*
Fluoroquinolone resistant parC and Fluoroquinolone resistant grrA	SA004 SA002	Fluoroquinolone antibiotic.	Antibiotic target alteration	sdrM	Is a drug transporter, responsible for increased resistance to antimicrobials such as norfloxacin
antibiotic-resistant isoleucyl-tRNA synthetase (ileS)	SA002	mupirocin	Antibiotic target alteration	Staphylococcus aureus mupA conferring resistance to mupirocin	Confers high level mupirocin resistance
Cfr 23S ribosomal RNA methyltransferase	SA002	Lincosamide antibiotic, oxazolidinone antibiotic, phenicol antibiotic, pleuromutilin antibiotic, streptogramin antibiotic	Antibiotic target alteration	cfrA	Encodes mutation 23SrRNA at A2503 by a methyltransferase
Rifamycin-resistant beta-subunit of RNA polymerase (rpoB)	SA002	Rifamycin antibiotics	Antibiotic target alteration Antibiotic target replacement	Staphylococcus aureus rpoB mutants conferring resistance to rifampicin	Confers high-level rifampin resistance

**TABLE 4:** Restriction enzymes detected in both SA002 and SA004.

Enzyme	Gene	Function		Recognition sequence
Type 1	*S.Sau8532II*	specificity subunit	block horizontal gene transfer between MRSA	AGGNNNNNGAT
	*M.SauTCHI*	methyltransferase		CCAYNNNNNNTGT
	*S.Sau20231II*	specificity subunit		CCAYNNNNNNTGT
Type IV	*SauUSI*	methyl-directed restriction enzyme	important barriers to the transfer of plasmid DNA from other bacteria	SCNGS

### Genomic characterization of the two isolates

WGS was used to characterize previously described AMR genes in both SA002 and SA004. Interestingly, restriction enzymes (Table 4) which are important for preventing gene transfer were detected (29). Both SA002 and SA004 had type I restriction enzymes which can block horizontal gene transfer between MRSA that are clinically important and type IV restriction enzymes which are important barriers to the transfer of plasmid DNA from other bacteria such as *Escherichia coli* (*E. coli*) (30). Important identified plasmids in SA002 were rep15 which carries resistance genes for tetracycline, clindamycin, trimethoprim, pristinamycin IIA, lincomycin, linezolid, chloramphenicol, florfenicol, tiamulin, mupirocin, ampicillin, cefoxitin, and gentamicin. The plasmid had an insertion sequence IS256 in the reverse strand and identity was 99.3% with an alignment coverage of 99.8%. Also, rep7a was detected and it carries resistance genes for tetracycline, lincomycin, clindamycin, pristinamycin IA, quinupristin, erythromycin, ampicillin, penicillin, and amoxicillin. The plasmid also carries virulence genes staphylokinase, gamma-hemolysin component B and C precursor, aureolysin, staphylococcal component inhibitor, and serine protease splA and B. The plasmid had an insertion sequence IS256 in the forward strand and identity was 100% with an alignment coverage of 100%. In SA004 important plasmids identified were rep10, which carries the resistance genes for trimethoprim, lincomycin, quinupristin, pristinamycin IA, erythromycin, penicillin, ampicillin, and amoxicillin. The plasmid also carries virulence genes staphylokinase and staphylococcal component inhibitor, and identity was 100% with an alignment coverage of 100%. Rep7a was also detected and it carries resistance genes for doxycycline, tetracycline, gentamicin, cefoxitin, ampicillin, and ampicillin + clavulanic acid. The plasmid also carries virulence genes leucocidin D component, gamma-hemolysin component B and C, aureolysin, serine protease splE, and identity was 100% with an alignment coverage of 100%. Both SA002 and SA004, based on top hits, had plasmids that showed similarity with plasmids identified in other clinically significant non-staphylococcal species and have also been detected in different geographical regions of the world such as pMP63C detected in *Morganella morganii*, pHVH-V1836-9 identified in *Enterococcus faecium* pIM13 identified in *Bacillus subtilis*, pK34-7-1 found in *Pseudomonas aeruginosa*, pK93G, and pT15G-1 found in *Staphylococcus lugdunensis*, The prediction of both isolates’ pathogenicity toward a human host, with gene identification set at high-level similarity with database entries (95-100%), matched important *S. aureus spp.* such as *S. aureus* subsp *aureus* USA300, known to be an epidemic clone of CA-MRSA, *S. aureus* subsp *aureus* str. Newman DNA, known to have robust virulence phenotypes, *S. aureus* subsp *aureus* NCTC 8325, known as the prototypical strain for genetic manipulation, *S. aureus* subsp *aureus* JH9, known to cause bacterial endocarditis and vancomycin-resistant *S aureus* infections (VRSA), *S. aureus* subsp *aureus* COL, known to cause MRSA infections, and *S. aureus* RF122, a major clone which causes severe bovine mastitis. The isolates also matched *S. aureus* strain MSSA476, known to have a protein msa (modulator of sarA) which enhances the expression of the staphylococcal accessory regulator (sarA) in a strain-dependent manner. sarA affects the transcription of accessory gene regulator (agr) and genes which encode virulence factors such as protein A (spa) and alpha toxin (hla). It also matched *S. aureus* subsp *aureus* JH1, known to cause bacterial endocarditis and MRSA infections, and *S. aureus* subsp *aureus* str. JKD6008 is known to cause both MRSA and VRSA infections.

## Discussion

This is a WGS-based study where we characterized two unique CA-MRSA out of 65 *S. aureus*, collected from 5 AMR surveillance sites in Kenya, which had novel STs 7460 and 7635. Recruited patients had SSTIs, which were community-onset as defined by the Centers for Disease Control and Prevention (CDC) distinguishing criteria for CA-MRSA from HA-MRSA (31). MRSA remains very clonal in the country with four main clonal complexes (5, 8, 22, and 30) (Figure 1) isolated from only 5 counties in the country out of 47 counties. CC8 to which novel STs 7460 and 7635 belong is the most successful MRSA clone in Kenya. Previously reported MRSA sequence types in Kenya to include STs 5, 8, 22, 30, 88, 152, 239, 241, 789, and 4705 belonging to four main clonal complexes 5, 8, 22, and 30 showing evolution within the complexes as shown in Figure 1. This information is important in medical practice because it allows antibiotic resistance studies to be done, which can guide the use of empirical treatment and can also be used to identify the emergence of new MRSA clones with increased resistance to antibiotics.

Antimicrobial susceptibility tests showed that the novel CA-MRSA STs had phenotypic resistance to cefoxitin, penicillin, erythromycin, clindamycin, ciprofloxacin, trimethoprim/sulfamethoxazole, and tetracyclines. Interestingly, linezolid and rifampin resistance was observed in SA002. Genotypic characterization revealed that linezolid resistance gene *cfr*, mupirocin resistance gene *mupA*, and rifampin resistance gene *rpoB* were present in SA002. Rifampin resistance gene had mutations that conferred high-level resistance to rifampin, a trait common in nosocomial *S. aureus* infections, which shows the spread of resistance between hospital and community settings, jeopardizing empirical treatment of community-onset infections which is very alarming. (32). Also, SA002 had AMR gene family rifamycin-resistant beta-subunit of RNA polymerase (rpoB) whose resistance mechanism is antibiotic target alteration and antibiotic target replacement. It also had an H481N mutation which confers high-level rifampin resistance. Mupirocin is not commonly used in Kenya as compared to linezolid, thus detecting resistance genes to mupirocin is a great concern, considering it is from a community-onset. This new resistance being detected from community isolates is a public health threat and also poses a serious challenge for clinicians in treating CA-MRSA infections as was also described by Singh et al. (33). This further highlights a wider spread of undetected genetically encoded resistance, which is a concern since most health facilities do not perform genotypic tests for antimicrobials during routine patient management. This suggests that there could be more genetically encoded resistance that has not been detected and documented, which may affect the effectiveness of antimicrobials, thus affecting treatment outcomes.

Notably, mutations were detected within *gyrA* genes, and plasmid-mediated *dfrB* and *dfrG* genes. Reports have indicated that the *dfrG* gene is increasingly being documented in Africa, and is associated with travelers, though more research needs to be done (34).

Genomic analysis revealed that novel ST 7460 had SCC*mec* IV 2B, which is classically associated with CA-MRSA, matching sample source which is from community-onset wound infection (i.e., SSTI). The new ST 7635 isolate had SCC*mec* type III (3a) that is classically associated with HA-MRSA, also from community-onset wound infection (SSTI), which shows evidence of the correlation between CA-MRSRA and HA-MRSA, where either one can be isolated from a community setting or hospital setting (35). Virulence factors observed showed several genes important in *Staphylococcus* survival in its hosts such as inhibiting both innate and adaptive immune responses, enhancing *Staphylococcus* pathogenicity, and production of bacterial anti-inflammatory agents and chemotaxis inhibiting protein. These include (i) *sbi*, which inhibits both innate and adaptive immune responses, (ii) *spls* which induce and enhance *Staphylococcus* pathogenicity, and (iii) *scn* plays a role in *Staphylococcus* host immune evasion and is also a staphylococcal component inhibitor, (iv) *hlg* known to have membrane damaging factors, (v) *luK* also known as bacterial invasins, attack natural killer cells, phagocytes, T-lymphocytes, and dendritic cells, (vi) *aur* which regulates biofilm growth cycle of *S. aureus*, (vii) *sak* known to activate plasminogen into plasmin which digests fibrin clots and activates latent matrix metalloproteinases leading to extreme proteolysis, (viii) *adsA* which evades host immune responses by modulating host pro-inflammatory responses, resulting in a prolonged infection, and (ix) *chp* a known bacterial anti-inflammatory agent and chemotaxis inhibiting protein.

Plasmids detected carried resistance genes coding for several antibiotics but most interestingly was plasmid rep15 in SA002 that had resistance genes to amphenicols, and linezolid (cfr gene) (expressed both phenotypically and genotypically) and mupirocin (mupA gene, expressed genotypically with no phenotypic break points in Vitek). Some of these antibiotics are not available in the country such as mupirocin, and this raises concern. Genetic characterization showed that the isolate had type I and type IV restriction enzymes that block gene transfer. The presence of a plasmid (rep15) containing resistance genes may suggest mutations affecting the functioning of these enzymes. Interestingly, AMR gene families; ATP-binding cassette (ABC) antibiotic efflux pump and major facilitator superfamily (MFS) antibiotic efflux pump that are multidrug efflux transporters and Cfr 23S ribosomal RNA methyl-transferase whose mode of action is antibiotic target alteration, could also play a role in linezolid resistance. Besides antibiotic efflux and 23S rRNA methylation mediated by the *cfr* gene, linezolid resistance may arise from point mutations in the 23S rRNA proteins L3, L4, and L22 as well as point mutations in domain V of the 23S rRNA gene. However, no mutations in the 23S rRNA were detected and an alignment of corresponding amino acid sequences for L3, L4, and L22 between SA002 and the USA300 reference showed no amino acid mutations. Perhaps this is the first report of linezolid resistance from Kenya, though it is quite likely the resistance might be existing undetected so far. Also, AMR gene family antibiotic-resistant isoleucyl-tRNA synthetase (ileS) whose mode of action is antibiotic target alteration and carries antimicrobial resistance ontology *Staphylococcus aureus* mupA conferring high-level resistance to mupirocin could play a role in mupirocin resistance (Table 3).

IS256 was detected in SA002 plasmids rep7a and rep 15 in the reverse and forward strands. Its implication clinically results in increased hypervirulence and cytotoxin production, and also heightened pathogenicity. Also, it results in to change in the promoter sequence of the repressor of toxins which is a master transcriptional regulator responsible for the expression of virulence factors in *S. aureus* (36).

The new resistance observed and the novel STs reported in this study highlight evolution within *S. aureus* and *S. aureus* infections, even though the evolution is not a parallel emergence from MSSA lineages but evolution within the clonal complex. The tree topology is in support of the classification that places ST 7460 and ST 7635 in CC8. The clinical significance posed further highlights the need for alternatives to antibiotics as the rate of resistance in hospital and community settings is superseding the rate of manufacturing new antibiotics. This is a clear indication that interventions need to be put in place. Also, molecular epidemiological studies are needed to reveal unidentified STs and clonal types, with key emphasis on genomic mutations that will have public health significance (e.g., the detection of IS256 in SA002, similar to that of USA500, and the clinical impact it carries) and identify new resistance to higher drugs such as linezolid and, mupirocin. Also, surveillance studies focusing on mutations within resistance genes with clinical relevance, emphasizing community settings are needed. Community isolates observed in this study seem to be showing resistance that has not been reported before in the country (e.g., mupirocin) and resistance that has only been reported before in hospital settings (e.g., rifampin). Infection prevention and control measures that are done in hospital settings need to be done in the community settings with the same measures to effectively prevent antimicrobial resistance spread between the two settings. Antimicrobial stewardship should focus on research of a cost-effective molecular method to include genotypic AMR tests in routine patient management that will not affect turnaround time and will give results promptly for better patient management.

## Conclusion

The appearance of linezolid resistance in SA002 adds to the increasing reports of linezolid resistance in different parts of the world. Due to excellent oral bioavailability, it is likely to be used for the management of outpatients with MRSA further creating pressure for the emergence of resistance. Linezolid is a reserved category antibiotic, much in need of preservation for serious clinical infections, and thus its resistance is important as the genotype observed was borne on a mobile element raising the possibility of rapid spread to other strains and/or species. Further, linezolid resistance could arise in settings of limited clinical use and should therefore be investigated from a One Health perspective. Delineation of HA-MRSA and CA-MRSA is becoming clinically challenging and molecular characterization helps to understand better the transmission dynamics of the pathogen. Data obtained provided preliminary insights into unidentified STs of CA-MRSA in Kenya. More studies in Africa need to be community-focused to reveal circulating strains and antimicrobial resistance that may have spread between hospital and community settings. Establishing AMR programs that enhance data sharing and information use, aimed at keeping track of new emerging resistance in hospital and community settings in Africa, need to be done. Novel strains detected have shown resistance to broad-spectrum anti-staphylococcal antibiotics not commonly used in the country, thus jeopardizing the successful treatment of MRSA infections. Given that most health facilities do not perform genotypic susceptibility tests for a routine patient management, genotypic resistance may go unnoticed in the continued absence of such programs.

## Data availability statement

The datasets presented in this study can be found in online repositories. The names of the repository/repositories and accession number(s) can be found in the article/supplementary material.

## Ethics statement

The studies involving human participants were reviewed and approved by Aga Khan University, Nairobi. Written informed consent to participate in this study was provided by the participants’ legal guardian/next of kin.

## Author contributions

GR and JNj: conceptualization. JNj and AM: methodology. GR, CN, and GO: supervision. JNj: writing – original draft. JNj, JNy, ZM, and GR: writing review and editing. All authors contributed to the article and approved the submitted version.

## Footnotes

1
https://cge.food.dtu.dk/services/

2
http://bacdb.cn/BacWGSTdb/

3
https://cge.food.dtu.dk/services/spaTyper/

4
https://spatyper.fortinbras.us/

5
https://spa.ridom.de/

6
https://cge.food.dtu.dk/services/PathogenFinder/

7
https://cge.food.dtu.dk/services/MobileElementFinder/

8
https://card.mcmaster.ca/analyze/rgi

9
https://bacdb.cn/BacWGSTdb

## References

[1] Tong SYC Davis JS Eichenberger E Holland TL Fowler VG. *Staphylococcus aureus* infections: epidemiology, pathophysiology, clinical manifestations, and management. *Clin Microbiol Rev.* (2015) 28:603–61. 10.1128/CMR.00134-14 26016486 PMC4451395

[2] Coates R Moran J Horsburgh MJ. Staphylococci: colonizers and pathogens of human skin. *Future Microbiol.* (2014) 9:75–91. 10.2217/fmb.13.145 24328382

[3] Becker REN Bubeck Wardenburg J. *Staphylococcus aureus* and the skin: a longstanding and complex interaction. *Skinmed.* (2015) 13:111–9. 26137737

[4] Stryjewski ME Corey GR. Methicillin-resistant *Staphylococcus aureus*: an evolving pathogen. *Clin Infect Dis.* (2014) 58(Suppl 1):S10–9. 10.1093/cid/cit613 24343827

[5] Bartels MD Worning P Andersen LP Bes M Enger H Ås CG Repeated introduction and spread of the MRSA clone t304/ST6 in northern Europe. *Clin Microbiol Infect.* (2021) 27:284.e1–284.e5. 10.1016/j.cmi.2020.05.004 32439595

[6] Jamrozy D Misra R Xu Z Ter-Stepanyan MM Kocharyan KS Cave R Novel methicillin-resistant *Staphylococcus aureus* CC8 clone identified in a hospital setting in armenia. *Front Microbiol.* (2019) 10:1592. 10.3389/fmicb.2019.01592 31354680 PMC6635598

[7] Laabei M Peacock SJ Blane B Baines SL Howden BP Stinear TP Significant variability exists in the cytotoxicity of global methicillin-resistant *Staphylococcus aureus* lineages. *Microbiology.* (2021) 167:001119. 10.1099/mic.0.001119 34928202 PMC8744995

[8] Lancashire JF Jones A Bergh H Huygens F Nimmo GR. Typing early Australian healthcare-associated MRSA: confirmation of major clones and emergence of ST1-MRSA-IV and novel ST2249-MRSA-III. *Pathology.* (2013) 45:492–4. 10.1097/PAT.0b013e3283632667 23856840

[9] Leme RCP Bispo PJM Salles MJ. Community-genotype methicillin-resistant *Staphylococcus aureus* skin and soft tissue infections in Latin America: a systematic review. *Braz J Infect Dis.* (2021) 25:101539. 10.1016/j.bjid.2021.101539 33607082 PMC9392117

[10] Schaumburg F Alabi AS Peters G Becker K. New epidemiology of *Staphylococcus aureus* infection in Africa. *Clin Microbiol Infect.* (2014) 20:589–96. 10.1111/1469-0691.12690 24861767

[11] Junie LM Jeican II Matroş L Pandrea SL. Molecular epidemiology of the community-associated methicillin-resistant *Staphylococcus aureus* clones: a synthetic review. *Med Pharm Rep.* (2018) 91:7–11. 10.15386/cjmed-807 29440945 PMC5808271

[12] Harris SR Feil EJ Holden MTG Quail MA Nickerson EK Chantratita N Evolution of MRSA during hospital transmission and intercontinental spread. *Science.* (2010) 327:469–74. 10.1126/science.1182395 20093474 PMC2821690

[13] Kyany’a C Nyasinga J Matano D Oundo V Wacira S Sang W Phenotypic and genotypic characterization of clinical *Staphylococcus aureus* isolates from Kenya. *BMC Microbiol.* (2019) 19:245. 10.1186/s12866-019-1597-1 31694531 PMC6836327

[14] Nyasinga J Omuse G John N Nyerere A Abdulgader S Newton M Epidemiology of <i&gt *Staphylococcus aureus* &lt/i> Infections in Kenya: current state, gaps and opportunities. *Open J Med Microbiol.* (2020) 10:204–21. 10.4236/ojmm.2020.104018

[15] Chesbrough M. *District Laboratory Practice in Tropical Countries, Part 2.* (2005). Available online at: https://books.google.co.ke/books (accessed Mar 6, 2022).

[16] CLSI. *Clinical & Laboratory Standards Institute: CLSI Guidelines - Performance Standards for Antimicrobial Susceptibility Testing.* Wayne, PA: CLSI (2018).

[17] Bolger AM Lohse M Usadel B. Trimmomatic: a flexible trimmer for Illumina sequence data. *Bioinformatics.* (2014) 30:2114–20. 10.1093/bioinformatics/btu170 24695404 PMC4103590

[18] Prjibelski A Antipov D Meleshko D Lapidus A Korobeynikov A. Using SPAdes De Novo Assembler. *Curr Protoc Bioinformat.* (2020) 70:e102. 10.1002/cpbi.102 32559359

[19] Gurevich A Saveliev V Vyahhi N Tesler G. QUAST: a quality assessment tool for genome assemblies. *Bioinformatics* (2013) 29:1072–5. 10.1093/bioinformatics/btt086 23422339 PMC3624806

[20] Simão FA Waterhouse RM Ioannidis P Kriventseva EV Zdobnov EM. BUSCO: assessing genome assembly and annotation completeness with single-copy orthologs. *Bioinformatics.* (2015) 31:3210–2. 10.1093/bioinformatics/btv351 26059717

[21] Jolley KA Bray JE Maiden MCJ. Open-access bacterial population genomics: BIGSdb software, the PubMLST.org website, and their applications. *Wellcome Open Res.* (2018) 3:124. 10.12688/wellcomeopenres.14826.1 30345391 PMC6192448

[22] Shopsin B Gomez M Montgomery SO Smith DH Waddington M Dodge DE Evaluation of protein a gene polymorphic region DNA sequencing for typing of *Staphylococcus aureus* strains. *J Clin Microbiol.* (1999) 37:3556–63. 10.1128/JCM.37.11.3556-3563.1999 10523551 PMC85690

[23] Alcock BP Raphenya AR Lau TTY Tsang KK Bouchard M Edalatmand A CARD 2020: antibiotic resistome surveillance with the comprehensive antibiotic resistance database. *Nucleic Acids Res.* (2019) 48:D517–25. 10.1093/nar/gkz935 31665441 PMC7145624

[24] Feng Y Zou S Chen H Yu Y Ruan Z. BacWGSTdb 2.0: a one-stop repository for bacterial whole-genome sequence typing and source tracking. *Nucleic Acids Res.* (2021) 49:D644–50. 10.1093/nar/gkaa821 33010178 PMC7778894

[25] Aiken AM Mutuku IM Sabat AJ Akkerboom V Mwangi J Scott JAG Carriage of *Staphylococcus aureus* in thika level 5 hospital, Kenya: a cross-sectional study. *Antimicrob Resist Infect Control.* (2014) 3:22. 10.1186/2047-2994-3-22 25057351 PMC4107749

[26] Omuse G Shivachi P Kariuki S Revathi G. Prevalence of panton valentine leukocidin in carriage and infective strains of &lti&gt*Staphylococcus aureus*&lt/i> at a Referral Hospital in Kenya. *Open J Med Microbiol.* (2013) 03:5–11. 10.4236/ojmm.2013.31002

[27] Tamura K Stecher G Kumar S. MEGA11: molecular evolutionary genetics analysis version 11. *Mol Biol Evol.* (2021) 38(7):3022–7. 10.1093/molbev/msab120 33892491 PMC8233496

[28] Kaatz GW McAleese F Seo SM. Multidrug resistance in *Staphylococcus aureus* due to overexpression of a novel multidrug and toxin extrusion (MATE) transport protein. *Antimicrob Agents Chemother.* (2005) 49:1857–64. 10.1128/AAC.49.5.1857-1864.2005 15855507 PMC1087643

[29] Roberts GA Houston PJ White JH Chen K Stephanou AS Cooper LP Impact of target site distribution for Type I restriction enzymes on the evolution of methicillin-resistant Staphylococcus aureus (MRSA) populations. *Nucleic Acids Res.* (2013) 41:7472–84. 10.1093/nar/gkt535 23771140 PMC3753647

[30] Monk IR. Genetic manipulation of Staphylococci-breaking through the barrier. *Front Cell Infect Microbiol.* (2012) 2:49. 10.3389/fcimb.2012.00049 22919640 PMC3417578

[31] Millar BC Loughrey A Elborn JS Moore JE. Proposed definitions of community-associated meticillin-resistant *Staphylococcus aureus* (CA-MRSA). *J Hosp Infect.* (2007) 67:109–13. 10.1016/j.jhin.2007.06.003 17669546

[32] Zhou W Shan W Ma X Chang W Zhou X Lu H Molecular characterization of rifampicin-resistant *Staphylococcus aureus* isolates in a Chinese teaching hospital from Anhui. China. *BMC Microbiol.* (2012) 12:240. 10.1186/1471-2180-12-240 23082766 PMC3485161

[33] Singh A Singh A Shukla S Agarwal L Chaturvedi P. Prevalence of mupirocin resistant *Staphylococcus aureus* isolates among patients admitted to a tertiary care hospital. *North Am J Med Sci.* (2014) 6:403. 10.4103/1947-2714.139293 25210674 PMC4158649

[34] Nurjadi D Olalekan AO Layer F Shittu AO Alabi A Ghebremedhin B Emergence of trimethoprim resistance gene dfrG in *Staphylococcus aureus* causing human infection and colonization in sub-Saharan Africa and its import to Europe. *J Antimicrob Chemother.* (2014) 69:2361–8. 10.1093/jac/dku174 24855123

[35] Kateete DP Bwanga F Seni J Mayanja R Kigozi E Mujuni B CA-MRSA and HA-MRSA coexist in community and hospital settings in Uganda. *Antimicrob Resist Infect Control.* (2019) 8:94. 10.1186/s13756-019-0551-1 31171965 PMC6547506

[36] Benson MA Ohneck EA Ryan C Alonzo F Smith H Narechania A Evolution of hypervirulence by an MRSA clone through the acquisition of a transposable element. *Mol Microbiol.* (2014) 93:664–81. 10.1111/mmi.12682 24962815 PMC4127135

